# Improvement in Artificial Dentures

**Published:** 1884-12

**Authors:** J. Taft


### THE DENTAL REGISTER.

VOL. XXXVIII.] DECEMBER, 1884. [No. 12.

### COMMUNICATIONS.

### Improvement in Artificial Dentures.

BY J. TAFT.

Read before the Ohio State Dental Society, Oct. 29, 1884.

In the insertion of artificial teeth, the object should be to restore, as far as possible, that which the loss of the natural teeth occasioned. Every one who has given attention to the subject knows the various purposes served by the natural teeth; notwithstanding this, the following is presented as a summary of the functions of these important organs:

First.—The teeth maintain the form, symmetry, and beauty of the face, and of the mouth in particular.

Second.—They aid in giving expression to the face in every variety of presentment. The various mental conditions and moods are usually clearly set forth upon the face, and this is especially true of the emotions and stronger operations of the mind.

Third.—The teeth are organs of speech; they control the voice and make distinct enunciation possible.

Fourth.—They facilitate the use of the soft parts, and serve to retain them in proper position and relation.

Fifth.—Mastication. Upon this function of the teeth is dependent, in a large measure, the welfare of the entire system. Especially is this true in reference to man, and, indeed, it may be said to be so of nearly all vertebrate animals. In mastication, the teeth serve for holding, tearing, cutting, and grinding.

These five particulars embrace the more important uses of the human teeth. They serve, in some animals, other purposes than these, but to these we need not now refer.

The loss of the teeth involves the impairment of all these functions, and the entire destruction of some of them. The preparation and insertion of artificial substitutes should have for its object the restoration of these functions to the highest degree possible. This will often, even after the best that art now affords has been done, fall far short of full restoration. In some cases and in some particulars this repair may be well nigh complete, while in others, total failure has been realized. How often is it that artificial teeth serve quite well for the purposes of mastication, while in appearance or expression of speech, or both, very imperfect results have been attained, sometimes making the case even worse than it was when wholly edentulous.

The experience of every dentist will illustrate this subject without further delineation. That the present practice of the profession does not meet all, nor even the larger portion of, the requirements is patent. Various circumstances contribute to this. The materials that have been so extensively, we might say almost universally, used for the last twenty-five years, have served very definitely to demoralize this branch of practice, and render far less perfect and efficient artificial substitutes than was aimed at prior to that time. The introduction of rubber, celluloid, and fusible metals for artificial dentures wrought much mischief to the higher art of this part of the profession. It rendered it so easy and simple to prepare a plate and place upon it artificial teeth, that the artistic features of the work were almost wholly overlooked. As artificial teeth were so easily produced, the expense attending their production was very greatly reduced, so much so as to preclude the expenditure of much time and skill in securing the best results. Too often the only point aimed at has been to insert something having the resemblance to teeth, with but little reference to their exact adaptability to the case, so that now, in the matter of appearance, almost every one who uses artificial teeth shows clearly at first glance that fact. This is many times shown—when the mouth is closed, by want of symmetry in the features of the face—the form, from the natural type, being so much changed by the loss of the teeth, and no effort made at restoration by the insertion of artificial teeth, that the defect is obvious to the most casual observer. Add to this the incongruous appearance of the teeth when the mouth is opened, and the case becomes repulsive to every one who has a just appreciation in this direction. Teeth, faulty in color, in form, in size, and in arrangement, render the face but little more tolerable than when without teeth altogether.

It is true that in all the materials mentioned, artistic work—that which shall be well nigh perfect in all these particulars—is possible; but not by the hasty methods that are generally employed. It is true, also, that in all dentures of the more difficult production, imperfect work may be done, but this style of work requires far more skill, time, and patience for its production in such form that it can be used at all, that all the particulars of the process are likely to receive more attention than in the more hastily produced kinds of work. There is a strong probability that ninety-five per cent of all the artificial dentures made in the profession is of the imperfect sort just referred to.

The great crying demand, then, in this matter of artificial dentures, is to improve all the methods we have, rather than to seek for some new device. New things come along in the regular order of development faster than me can utilize them, and they have in some instances been a disadvantage in that, by them the attention has been diverted from modes and methods the value of which has been fully established by long usage. It is an almost universal fault that we are more anxious to find and learn new things, than to improve and perfect those that we have. I have but little to say in respect to particular modes of work, but I am fully impressed with the fact that a return to some of the former methods of producing artificial substitutes is an end greatly to be desired. Perhaps not more than one in ten of the profession in this State is prepared or has the ability to construct artificial dentures upon gold, continuous gum, or porcelain. The profession in the aggregate would certainly make a great gain should all its members, or even a large proportion of them, become able to use well all the methods of inserting artificial teeth, so that the selection might be made best adapted to each and every case presented. No two cases require exactly the same method of procedure.

Not much that is really new has been presented to the profession for many years; no new principles have been proposed, and yet modes of applying and adapting have been introduced and pressed upon the attention of the profession, that ought to be well tested and well considered before general adoption. I refer now more particularly to that form of work known as "Bridge Work," in which the teeth are placed upon a band or bar of gold or other material, that stretches across spaces of greater or less extent, where teeth have been removed altogether, or attachments made to sound teeth or to the roots of teeth that have been decayed, the crowns of which have been removed. In these dentures no plate rests upon the gum, the entire support and attachment of the denture being made to natural teeth or roots, these attachments usually embracing a large share, and in many cases the entire crown of the tooth or teeth. These attachments are usually made with a view to remain permanently. This is a method of exceedingly doubtful propriety. In some cases which have come under my notice, teeth that were perfect before the operation, have been cut and injured so as to greatly facilitate decay. The drilling, grooving, and slotting in sound teeth for the purpose of receiving such fixtures can hardly be too strongly condemned; and even when this is not done, the application of caps or bands that fully cover the crown or crowns of teeth, will oftentimes be the occasion of early decay. In some cases the pressure and straining of such dentures upon sound teeth, produces periostial disturbance that results in a chronic inflammatory condition, and sometimes alveolar abscesses. This method of inserting teeth will certainly require much modification before it can receive the unqualified approval of the more intelligent of the profession.

A very great stride has been made in the last few years in the attachment of artificial teeth to the roots of teeth. Various methods have been developed to a very great degree of efficiency, and most of these are well worth attention and employment.

Of the various methods now before the profession it would be difficult to find a good root of a tooth upon which a useful and comfortable crown can not be inserted. With these various methods it is important that every member of the profession should become familiar, in order that the largest sphere of usefulness may be served by their application. In closing these remarks, I can not, perhaps, do better than to emphasize the thought that perfecting the modes and processes now in use is far more important than the discovery of new devices; not that the latter should be discouraged, but that the former should receive the attention they deserve.

